# Deep learning in Myocarditis: A novel approach to severity assessment

**DOI:** 10.1371/journal.pone.0354714

**Published:** 2026-08-12

**Authors:** Makoto Nishimori, Tomoyuki Otani, Yasuhide Asaumi, Keiko Ogo, Yoshihiko Ikeda, Kisaki Amemiya, Teruo Noguchi, Chisato Izumi, Masakazu Shinohara, Kinta Hatakeyama, Kunihiro Nishimura

**Affiliations:** 1 Division of Molecular Epidemiology, Kobe University Graduate School of Medicine, Kobe, Japan; 2 Department of Preventive Medicine and Epidemiology, National Cerebral and Cardiovascular Center, Osaka, Japan; 3 Department of Pathology, National Cerebral and Cardiovascular Center, Osaka, Japan; 4 Department of Cardiology, National Cerebral and Cardiovascular Center, Osaka, Japan; Universitas Muhammadiyah Aceh, INDONESIA

## Abstract

**Background:**

Myocarditis is life-threatening in the acute phase, yet biopsy—the diagnostic gold standard—lacks an objective method to quantify cardiomyocyte damage. We developed deep learning models to derive a pathology-based severity index for myocarditis from whole-slide biopsy images.

**Methods and results:**

We retrospectively analyzed 305 consecutive patients (1,056 digitized hematoxylin–eosin slides) who underwent endomyocardial biopsy between 2002 and 2021 at the National Cerebral and Cardiovascular Center; 145 met Dallas criteria for myocarditis and were used for severity modeling. Severe myocarditis was defined by short-term in-hospital outcomes (SCAI-aligned cardiogenic shock, initiation of mechanical circulatory support, or death). A multiple instance learning (MIL) classifier was first trained on slide-level myocarditis labels. We then built two severity models: (1) logistic regression using lymphocyte density derived from a YOLOv8-based object detector (Model 1), and (2) a Transformer that processed the top MIL-ranked patches to predict severe versus non-severe myocarditis (Model 2). Model 1 confirmed a strong association between inflammatory burden and severe outcomes (AUROC 0.809). Model 2 achieved superior discrimination (AUROC 0.993) with higher accuracy and precision. Attention maps indicated that Model 2 focused not only on inflammatory infiltrates but also on myocyte injury and architectural disruption, suggesting broader histologic signal capture. The final output was a continuous pathology-based severity score; clinical variables were not input to the models.

**Conclusions:**

Combining MIL with a Transformer enables comprehensive extraction of histologic features associated with clinically severe myocarditis and yields an objective, reproducible tissue-injury index. This score is intended to standardize histologic severity assessment and complement, rather than replace, clinical evaluation; incremental clinical utility requires prospective, multi-center validation.

## Introduction

Myocarditis is a very serious and high-risk disease carrying the risk of circulatory fluctuations during the acute phase [1–3]. Although clinical assessment typically involves symptoms, laboratory findings, and electrocardiograms, the definitive diagnosis relies on histopathological evaluation of myocardial biopsy [4]. Early assessment of clinical disease severity is also essential for guiding treatment and estimating prognosis [5].

Although a study evaluating the clinical severity of myocarditis from histopathology has been reported [6,7], an accurate index for evaluation has not yet been established. A factor that histologically defines the severity of myocarditis is the degree of myocardial cell damage. However, there is no method for morphologically objective assessment of cardiomyocyte damage, such as coarsening of the myocyte cytoplasm, irregular shape, or disorganization of the arrangement of myocytes. Therefore, we aimed to develop an artificial intelligence (AI)-based model capable of detecting myocardial tissue features indicative of clinical disease severity.

In previous studies on pathology AI models, various deep learning models have been developed for analyzing pathological tissues, including myocarditis [8–11]. While AI models for diagnosing myocarditis have been reported, no models specifically designed to assess the clinical severity of myocarditis have been developed. Achieving this goal requires a model capable of comprehensively interpreting whole-slide pathology images (WSIs). However, these images tend to be extremely large, making direct input into the model inefficient in terms of both computational resources and cost. Multiple Instance Learning (MIL) [12] provides a potential solution by dividing slides into smaller regions for training. In addition, MIL does not require detailed pixel-level annotations, instead using slide-level labels, which is particularly advantageous for pathology applications [13]. Transformer architectures [14]—now widely used as the backbone of large language models—have recently demonstrated high learning efficiency across multiple domains. To date, there are no reports of combining MIL with Transformer-based methods for myocardial pathology. We anticipate that this combination could significantly enhance performance in analyzing myocardial tissue. Therefore, the objective of this study is to develop a high-accuracy AI model capable of comprehensively interpreting WSIs to evaluate the clinical severity of myocarditis, ultimately aiming for clinical applicability.

We sought to derive and internally validate a pathology-based severity index for myocarditis from routine whole-slide images. Because no validated histopathologic severity scale exists beyond inflammatory cell counts, we used short-term clinical severity (SCAI-aligned cardiogenic shock/MCS/death) only as an outcome-aligned training signal to establish criterion validity. This study does not propose a stand-alone clinical decision-support tool; rather, it develops an objective tissue-injury indicator that could be standardized across cohorts and later combined with hemodynamics and imaging in multimodal models.

## Methods

### Study design, data sources, and patient population

**Fig 1:**
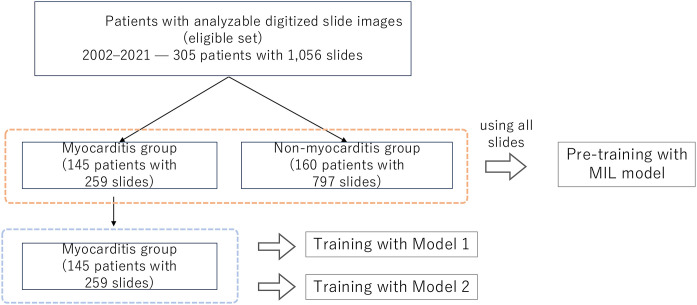
Study cohort and analysis workflow. The flowchart shows the selection and analysis process for patients undergoing myocardial biopsy from 2002 to 2021. From 305 eligible patients (1,056 slides), 145 biopsy-proven myocarditis cases (259 slides) and 160 without myocarditis (797 slides) were identified. The myocarditis group was used for training two models after pre-training with a MIL model.

We included 305 consecutive patients for whom at least one digitized H&E whole-slide image (WSI) of the index endomyocardial biopsy was available between 2002–2021 (1,056 WSIs). Digitization was part of routine archiving/batch scanning and independent of clinical outcomes. Within this eligible set, 145 had biopsy-proven myocarditis and 160 did not; the myocarditis cohort was used for severity-score modeling. Non-myocarditis cases were predominantly post–heart-transplant surveillance biopsies showing no acute cellular rejection (ISHLT ACR 0R) (Fig 1). In this cohort, biopsies were generally performed on the day of or after admission.

### Ethics approval

This study was approved by the Ethics Committee of the National Cerebral and Cardiovascular Center (NCVC, Approval No. R21078-2) and conducted in accordance with the Declaration of Helsinki. The requirement for individual informed consent was waived by the committee based on an opt-out policy. De-identified (pseudonymized) data were accessed for research purposes on 9 June 2022. The authors could not access identifiable information; the re-identification key was retained by NCVC and was not provided to the authors.

### Diagnostic and severity criteria for myocarditis

Diagnosis of myocarditis was determined by three cardiac pathologists based on the comprehensive Dallas criteria with clinicopathologic correlation.

Severe myocarditis was defined a priori by short-term in-hospital outcomes: (i) cardiogenic shock (SCAI stages C–E; operationalized as sustained hypotension <90 mmHg for ≥30 min or the need for vasoactive/inotropic agents to maintain MAP ≥ 65 mmHg, plus ≥1 sign of hypoperfusion such as lactate ≥2.0 mmol/L, oliguria, cool extremities, or altered mentation), (ii) initiation of temporary MCS (IABP, Impella, VA-ECMO, or temporary VAD), or (iii) in-hospital death [15].

Intended use of the model output. The model produces a continuous pathology-based severity score (higher values indicate greater tissue injury). Clinical variables were not provided to the model, and the score is not intended for bedside decision-making in this study. The outcome labels were used solely to align the score with clinically recognized severity and to test its criterion validity.

### Whole slide image data

Hematoxylin and eosin-stained slides were digitized using a Hamamatsu NanoZoomer S210 C13239 series at a Source lens magnification of 40x, capturing detailed virtual slide images.

### Data preprocessing

We used the openslide (Python) library [16] to read and process each ndpi file. To accommodate the large size of whole-slide images (WSIs) while retaining detailed histological features, we segmented each slide into 256 × 256-pixel patches. These dimensions were chosen based on preliminary experiments and prior pathology studies, which suggested a balance between sufficient tissue context and computational feasibility [17]. Patches containing less than 50% tissue (based on simple thresholding of non-background pixels) were excluded to reduce noise from empty or artifact-laden regions. Data augmentation (e.g., random horizontal/vertical flips, rotations, and slight color jitter) was applied to mitigate overfitting and improve the model’s robustness to variations in staining and tissue orientation. [18]

### Pretraining model

To efficiently extract features indicative of myocarditis, a pretraining model was developed using all cases. The cases were randomly divided at the patient level into training (70%), validation (15%), and test datasets (15%); all WSIs from a given patient were assigned exclusively to one partition to prevent slide-level data leakage. A MIL model was employed, which does not require pixel-level annotations but uses slide-level labels for presence or absence of myocarditis.

### Model developement and training

**Fig 2:**
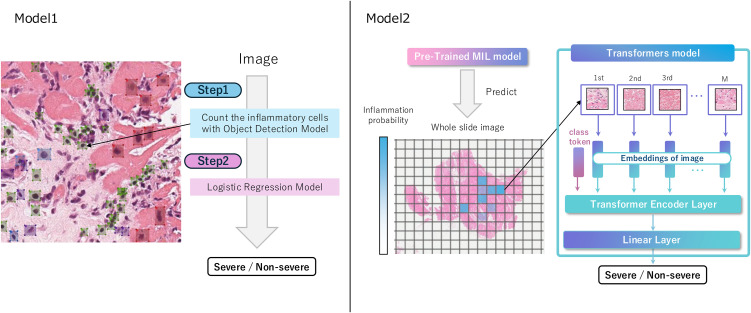
AI Models for severity classification. The figure shows two AI models used to classify myocarditis severity. Model 1: An object detection AI model counts inflammatory cells, followed by a logistic model to classify severity as severe or non-severe. Model 2: A trained MIL model predicts myocarditis probability. The MIL features are processed through a Transformer model to provide the final diagnosis of severe or non-severe.

In this study, we developed two distinct approaches for severity prediction, each leveraging a different methodology. We then evaluated and compared their prognostic performance to determine which approach could more accurately predict clinical outcomes (Fig 2). For myocarditis severity modeling, the myocarditis cohort was also partitioned at the patient level so that no patient's slides appeared in more than one dataset.

### Model1 (Inflammatory cell detection and logistic regression)

We focused on quantifying inflammatory infiltration as an initial, straightforward indicator of disease severity. First, two board-certified pathologists annotated lymphocytes on randomly selected patches. Lymphocytes were defined as mononuclear inflammatory cells with round nuclei, dense chromatin, and scant cytoplasm. All annotations were based on the consensus of the two pathologists. Using these annotations, we trained an object detection model based on YOLOv8 model [19] to count inflammatory cells per unit tissue area. A total of 1,000 annotated images were used; these were split into training (70%), validation (15%), and test (15%) sets. Default YOLOv8 hyperparameters were employed. We evaluated the object detection performance using metrics such as mean average precision on the validation set. The resulting feature for each 256 × 256 patch was the average lymphocytes count, computed by summing YOLOv8 detections and normalizing by tissue area.

Finally, we applied a univariate logistic regression model, using the lymphocytes density as the sole independent variable to predict disease severity. This univariate approach enabled us to isolate the contribution of lymphocytes infiltration, with future plans to incorporate additional factors for more comprehensive modeling.

### Model2 (Transformer-based severity classification)

Model 2 employed a Transformer-based approach to classify disease severity. We began by applying a pretrained MIL model to estimate the likelihood of myocarditis for each patch. To reduce computational demands and focus on the most informative regions, we selected the top N patches showing the highest probabilities of myocarditis. These patches were then encoded into embedding vectors and processed by a Transformer encoder along with a Class Token. The final classification layer—a multi-layer perceptron applied to the Class Token output—performed a binary classification (severe vs. non-severe) via a sigmoid function. All major hyperparameters, including the Transformer architecture (e.g., number of layers, attention heads), the learning rate, and batch size, were optimized using Bayesian optimization [20]. Training was conducted in parallel on two workstations, each equipped with an NVIDIA GeForce RTX 4090 GPU, to expedite processing. Validation set performance guided hyperparameter tuning, ensuring the model converged effectively on the classification task.

### Decision rationale visualization

To elucidate the contributions of different regions to the severity diagnosis, we visualized the attention scores assigned to each patch by the Class Token in the Transformer model [14], indicating key areas impacting the severity assessment (Fig 5).

**Fig 3:**
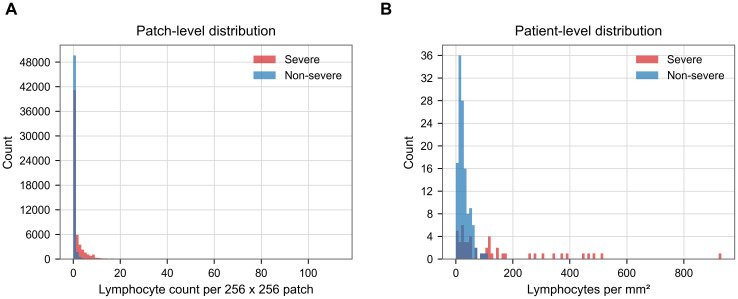
Distribution plot of lymphocytes cells detection model. (A) The distribution of lymphocytes counts per patch, comparing severe and non-severe samples. The x-axis represents the number of lymphocytes per patch, and the y-axis represents the frequency of these counts. (B) The distribution of lymphocytes counts per 1 mm² for each patient, comparing severe and non-severe samples. The x-axis represents the number of lymphocytes per 1 mm², and the y-axis represents the frequency of these counts.

**Fig 4:**
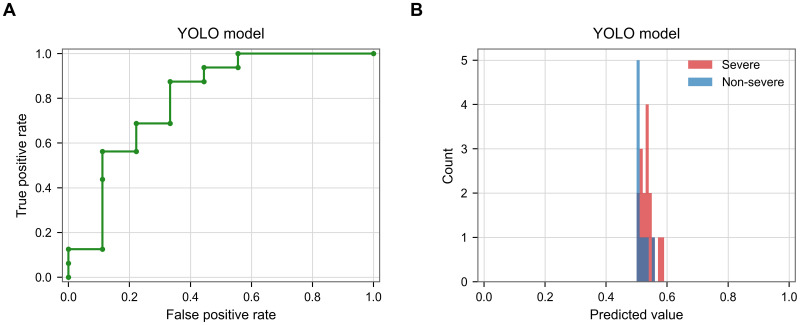
Results of Model 1. (A) Receiver Operating Characteristic (ROC) curve for Model 1. The x-axis represents the false positive rate, and the y-axis represents the true positive rate. Each point on the curve represents a different threshold value used to discriminate between classes. (B) Histogram of predicted values for Model 1. The x-axis represents the predicted value, and the y-axis represents the count of predictions. The red bars indicate positive samples, and the blue bars indicate negative samples.

### Statistics

Model performance was assessed through precision, recall, F1-score, area under the receiver operating characteristic curve (AUROC), and area under the precision-recall curve (AUPRC). Overall accuracy was also evaluated. Point estimates were calculated on the held-out test set, and 95% confidence intervals were estimated using the bootstrap method [21].

### Software

The analysis was conducted using Python 3.8, with PyTorch 1.8 [22] for deep learning tasks and scikit-learn for statistical calculations.

## Results

### Patients characteristics

The study period spanned from January 2002 to December 2021 at the NCVC. A total of 145 patients diagnosed with myocarditis, including children, were eligible for inclusion. These patients had undergone myocardial biopsy and were confirmed to have myocarditis. No specific exclusion criteria were set.

The average age of these patients was 45 years (±21.46 years), with 59% being male. Among the total cohort, 88 patients (60.7%) were classified as severe cases. The average peak creatine kinase-MB values were 135.43 for the entire cohort, 166.83 for the severe group, and 54.99 for the non-severe group. Detailed information on other laboratory data at the onset for each group is provided in Table 1.

**Table 1:** Patient characteristics.

	All myocarditis patients (n = 145)	Severe (n = 88)	Non-severe (n = 57)
Age (years)	45.00 (21.46)	45.23 (20.50)	44.65 (23.04)
Men, n (%)	86 (0.59)	51 (0.58)	35 (0.61)
Women, n (%)	59 (0.41)	37 (0.42)	22 (0.39)
Transplant, n (%)	5 (0.03)	5 (0.06)	0 (0.00)
Cardiac death, n (%)	17 (0.12)	17 (0.19)	0 (0.00)
Shock, n (%)	64 (0.44)	64 (0.73)	0 (0.00)
Cardiac arrest, n (%)	10 (0.07)	10 (0.11)	0 (0.00)
Mechanical support, n (%)	79 (0.54)	79 (0.90)	0 (0.00)
IABP, n (%)	50 (0.34)	50 (0.57)	0 (0.00)
Impella, n (%)	21 (0.14)	21 (0.24)	0 (0.00)
ECMO, n (%)	67 (0.46)	67 (0.76)	0 (0.00)
VAD, n (%)	40 (0.28)	40 (0.45)	0 (0.00)
Bad prognosis, n (%)	88 (0.61)	88 (1.00)	0 (0.00)
Steroid, n (%)	67 (0.46)	47 (0.53)	20 (0.35)
Catecholamine, n (%)	97 (0.67)	79 (0.90)	18 (0.32)
SBP on admission (mmHg)	102.00 (22.22)	93.82 (19.59)	115.58 (19.64)
HR on admission (beats/min)	91.35 (26.51)	94.78 (25.38)	86.02 (27.58)
LVDd on admission (mm)	49.67 (10.07)	47.23 (9.04)	53.57 (10.48)
%FS on admission (%)	16.52 (11.22)	13.92 (11.03)	21.15 (10.11)
PWT on admission (mm)	9.89 (2.68)	10.26 (2.81)	9.24 (2.34)
Peak LVDd (mm)	53.75 (9.31)	52.09 (8.74)	56.65 (9.64)
Peak %FS (%)	12.50 (9.11)	9.56 (7.99)	18.24 (8.50)
Peak PWT (mm)	10.92 (2.91)	11.55 (2.94)	9.71 (2.45)
Peak CK-MB (IU/L)	135.43 (239.87)	166.83 (269.73)	54.99 (101.71)
Peak troponin (assay-specific units)	6.05 (12.57)	8.14 (14.66)	1.62 (3.27)
Peak AST (IU/L)	832.05 (2444.32)	1282.45 (3010.97)	84.40 (97.62)
Peak ALT (IU/L)	395.19 (898.82)	589.02 (1090.07)	73.42 (136.04)
Cr on admission (mg/dL)	1.23 (1.18)	1.25 (1.02)	1.22 (1.42)
Peak Cr (mg/dL)	1.65 (1.81)	1.71 (1.49)	1.56 (2.27)

The data are presented as the mean (SD) for normally distributed data and as the median and interquartile range for non-normally distributed data; n (%), IABP, intra-aortic balloon pump; ECMO, extracorporeal membrane oxygenation; VAD, ventricular assist device; SBP, systolic blood pressure; HR, heart rate; LVDd, left ventricular end-diastolic dimension; %FS, fractional shortening percentage; PWT, posterior wall thickness; CK-MB, creatine kinase-MB; AST, aspartate aminotransferase. Troponin values are reported in assay-specific units.

### Pre-training with multiple instance learning model

A pre-training model was developed using MIL on a dataset of 305 patients with 1,056 myocardial pathology samples, including myocarditis patients, to diagnose myocarditis (Table 2). The results from the training dataset showed an accuracy of 0.751 and an AUROC of 0.717. In the validation dataset, the model achieved an accuracy of 0.749 and an AUROC of 0.712. Visualization of the diagnostic rationale in the inference phase was performed, with heatmaps illustrating the areas diagnosed as myocarditis shown in S1 Fig. The heatmaps indicate higher values in regions where inflammatory cells are more concentrated.

**Table 2:** Results of pre-training model.

	Training	Validation
Accuracy	0.751	0.749
Recall	0.387	0.479
Precision	0.593	0.561
F1-Score	0.468	0.517
AUROC	0.717	0.712
AUPRC	0.586	0.583

The table presents the performance metrics of the pre-training model during the training and validation phases. The metrics include accuracy, recall, precision, F1-Score, AUROC (Area Under the Receiver Operating Characteristic curve), and AUPRC (Area Under the Precision-Recall Curve).

### Model 1: Predicting myocarditis severity using a deep learning model based on inflammatory cell infiltration

An object detection model (YOLOv8) was pre-trained to detect lymphocytes, based on annotations by two myocardial pathology specialists. This model was used to automatically detect the number of lymphocytes, and a logistic regression model was constructed using the number of lymphocytes per unit area as a variable to evaluate clinical myocarditis severity. The results of inflammatory cell detection by the object detection model are shown in S1 Fig. Histogram plots of lymphocyte counts per 256 × 256-pixel patch showed that clinically severe myocarditis cases had relatively higher lymphocytes counts per patch, while non-severe cases had lower counts (Fig 3A). When converted to the number of lymphocytes per unit area (1 mm²), high values were observed in clinically severe cases, but most overlapped with non-severe cases (Fig 3B). The logistic regression model using the number of lymphocytes per unit area as a variable resulted in an AUROC of 0.809 (Fig 4A). Histograms of the predicted values for clinically severe and non-severe cases were also shown (Fig 4B).

### Model 2: Predicting myocarditis severity using a transformer-based model

**Fig 5:**
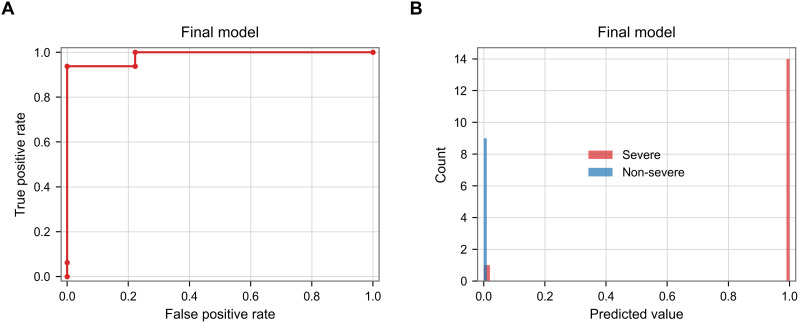
Results of Model 2. (A) Receiver Operating Characteristic (ROC) curve for Model 2. The x-axis represents the false positive rate, and the y-axis represents the true positive rate. Each point on the curve represents a different threshold value used to discriminate between classes. (B) Histogram of predicted values for Model 2. The x-axis represents the predicted value, and the y-axis represents the count of predictions. The red bars indicate positive samples, and the blue bars indicate negative samples.

The pre-trained MIL model was fixed, and for each patch, the MIL model inferred the presence of myocarditis. The top N candidate patches were then input into a Transformer-based model to predict clinical myocarditis severity. The results showed an AUROC of 0.993 (0.952–1.000), an accuracy of 0.92 (0.800–1.000), a precision of 1.000 (1.000–1.000), and a recall of 0.875 (0.688–1.000) (S1 Table). ROC curves and histograms of predicted values are shown in Fig 5-A and 5-B.

### Comparison of models for predicting myocarditis severity

The performance summary for predicting clinically severe myocarditis is shown in S1 Table. Model 2 showed higher accuracy, precision, AUROC, and AUPRC than Model 1, although recall was lower.

### Visualization of rationale for severity prediction

**Fig 6:**
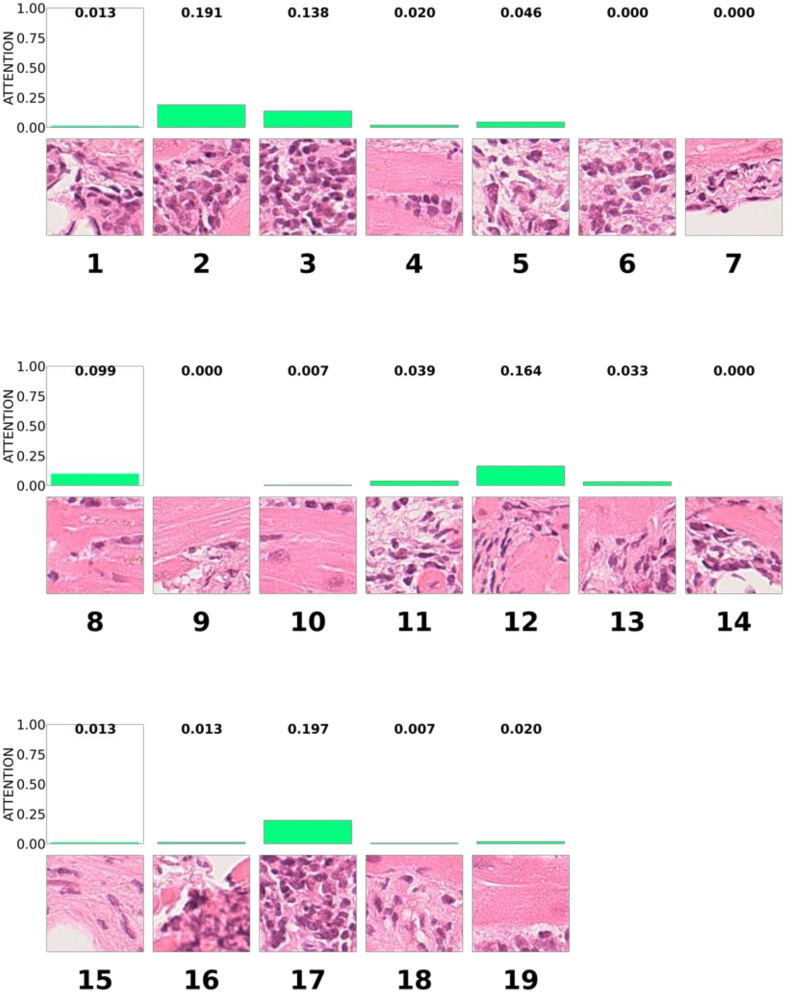
Attention scores for severe myocarditis. The figure displays attention scores assigned to histological images of severe myocarditis by the model. Each image is associated with an attention score, indicating the model's focus on different regions of the images. The green bars above each image represent the attention score, with higher bars indicating greater attention by the model.

To visualize the rationale for clinical severe myocarditis prediction with Model 2, the attention scores of each input patch were illustrated for representative cases (Fig 6).

## Discussion

In this study, we developed two AI models to evaluate the severity of myocarditis. Model 1, based on lymphocytes density, achieved an AUROC of 0.809, providing novel evidence that lymphocytes count correlate strongly with disease severity. In contrast, Model 2, which uses a Transformer-based approach to analyze whole-slide pathology images, attained a higher AUROC of 0.993. These findings suggest that a deep learning model capable of integrating diverse morphological features throughout the tissue may outperform a simpler model focused solely on the extent of inflammatory cell infiltration.

Our primary contribution is an objective, reproducible histopathologic indicator of tissue injury in myocarditis. Prior work has centered on inflammatory-cell counts, whereas myocyte injury and architectural derangement lack standardized quantification. A pathology-based index can (i) standardize severity grading across centers and time, enabling comparable cohorts and trials; (ii) serve as a research anchor to relate tissue patterns to virology, immunophenotypes, or genomics; and (iii) be combined with clinical variables in future multimodal risk models. Consistent with this scope, the present study does not claim that pathology alone should guide acute treatment decisions; rather, it adds a missing histologic dimension that complements hemodynamics and imaging.

The clinical significance of Model 1 lies in its demonstration that inflammatory cell density is closely linked to disease severity. While immunological mechanisms have long been implicated in myocarditis [23], research quantifying inflammatory infiltrates as a direct prognostic marker remains sparse. One plausible reason for the superior performance of Model 2 is that clinically severe myocarditis represents a composite histopathologic phenotype that cannot be captured by inflammatory-cell burden alone. Histopathologically, severe cases may show not only dense inflammatory infiltrates but also myocyte injury, interstitial change, and architectural disruption. Computationally, the Transformer-based Model 2 can integrate spatially distributed and interrelated information across multiple informative patches; unlike recurrent neural network (RNN)-based methods [24], this approach can capture broader contextual information rather than collapsing each case into a univariate summary. Consistent with this interpretation, the attention maps suggested that Model 2 focused not only on inflammatory infiltrates but also on regions with myocyte injury and tissue disorganization.

### Limitations

Several limitations must be acknowledged. First, this is a retrospective, single-center analysis and therefore cannot establish causality. Second, external validation was not performed, so the generalizability of these models across institutions, scanners, staining protocols, practice eras, and specific myocarditis subtypes (such as giant cell myocarditis) remains uncertain. Third, the long inclusion period and modest sample size may have introduced temporal heterogeneity and limit the robustness of the reported performance estimates.

## Conclusion

We introduce a pathology-based severity index for myocarditis that aligns with short-term clinical severity. The index offers a standardized histologic indicator of tissue injury and complements clinical assessment, providing a foundation for future multimodal prognostic modeling. Future studies should evaluate whether integrating this pathology-based severity score with clinical variables, hemodynamic measures, biomarkers, and cardiac imaging further improves prognostic performance. Prospective, multi-center validation will be necessary to determine clinical utility and the potential role of such multimodal models in decision-support settings.

### Code availability

The author-generated scripts used in this study are publicly available at GitHub and archived on Zenodo (https://doi.org/10.5281/zenodo.19370751). Trained model weights are not included in the public repository in the current revision, but may be made available for research purposes upon reasonable request and subject to institutional and ethical approval.

## Supporting information

S1 Fig Model-derived myocarditis probability maps. (A) Biopsy with high predicted probability (dark green); magnified H&E confirms dense lymphocytic infiltration. (B) Biopsy with low probability; enlarged view shows preserved cardiomyocytes and minimal inflammation. Scale bars = 100 µm.
(PDF)

S1 Table Detailed performance metrics of Models 1 and 2. The table compares the performance of two models (Model 1 and Model 2) in predicting the severity of myocarditis. Metrics include accuracy, precision, recall, AUROC (Area Under the Receiver Operating Characteristic curve), and AUPRC (Area Under the Precision-Recall Curve). Point estimates and 95% confidence intervals (CI) estimated by bootstrap are provided for each metric.
(XLSX)

## Abbreviations

AI: Artificial Intelligence
MIL: Multiple Instance Learning
WSIs: Whole-Slide pathology Images
AUROC: Area Under the Receiver Operating Characteristic curve
AUPRC: Area Under the Precision-Recall Curve
RNN: Recurrent Neural Network
