# Supplementary material for: Deep learning in Myocarditis: A novel approach to severity assessment
Source: PLoS One. 2026 Aug 12;21(8):e0354714. doi: 10.1371/journal.pone.0354714 (PMC13465861; doi:10.1371/journal.pone.0354714)
Supplement: Supplementary file 1 [file pone.0354714.s001.pdf]

Supplemental Figure1: Model-derived myocarditis probability maps.

A

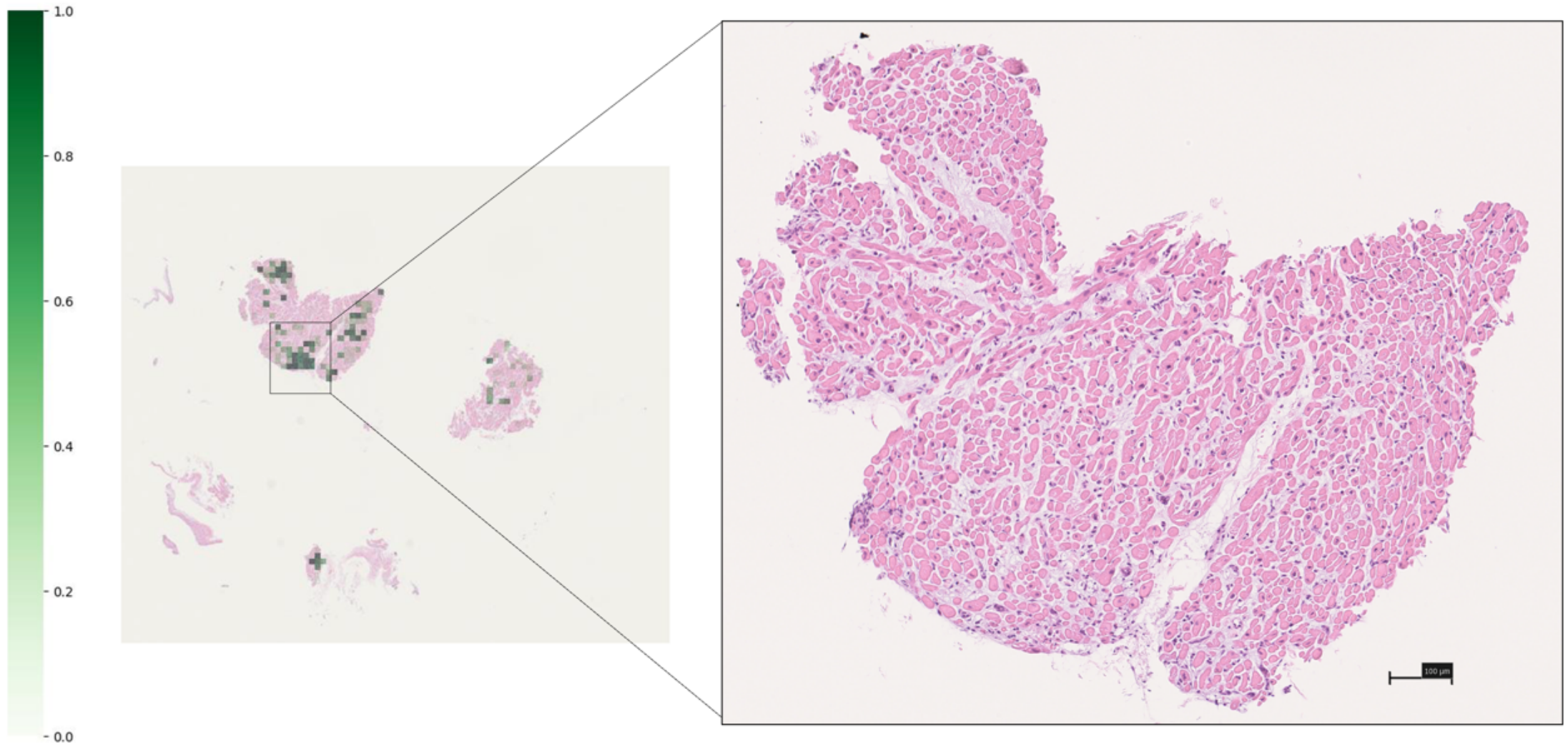

B

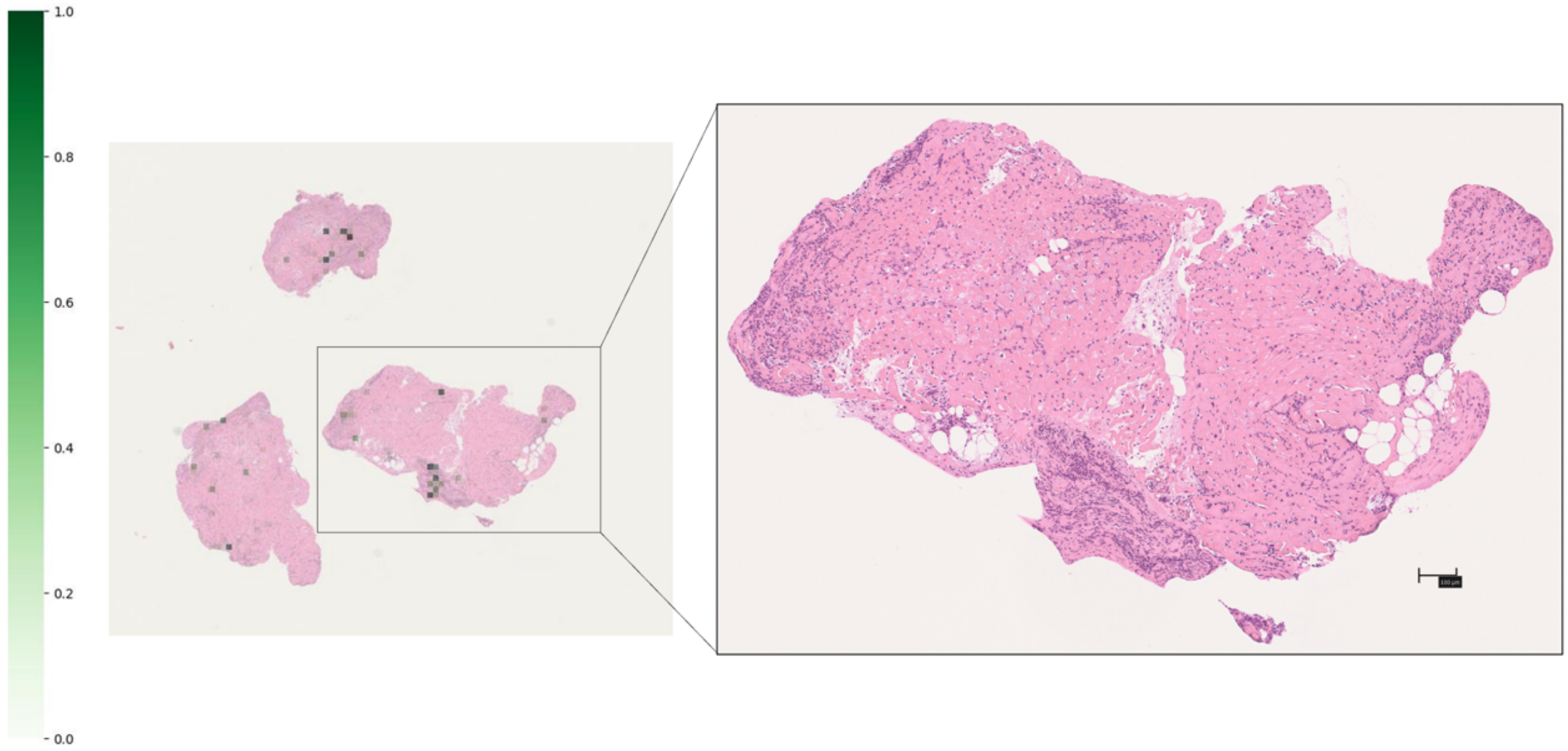

(A) Biopsy with high predicted probability (dark green); magnified H&E confirms dense lymphocytic infiltration. (B) Biopsy with low probability; enlarged view shows preserved cardiomyocytes and minimal inflammation.

Scale bars = 100 µm.
